# Therapeutic effect of intravesical administration of paclitaxel solubilized with poly(2-methacryloyloxyethyl phosphorylcholine-co-*n*-butyl methacrylate) in an orthotopic bladder cancer model

**DOI:** 10.1186/s12885-015-1338-2

**Published:** 2015-04-26

**Authors:** Koetsu Tamura, Eiji Kikuchi, Tomohiro Konno, Kazuhiko Ishihara, Kazuhiro Matsumoto, Akira Miyajima, Mototsugu Oya

**Affiliations:** 1 Department of Urology, Keio University School of Medicine, 35 Shinanomachi, Shinjuku-ku, Tokyo 160-8582 Japan; 2 Department of Bioengineering and Materials Engineering, School of Engineering, The University of Tokyo, 7-3-1 Hongo, Bunkyo-ku, Tokyo 113-8656 Japan

**Keywords:** Urinary bladder, Urinary bladder neoplasms, Intravesical, Paclitaxel

## Abstract

**Background:**

To evaluate the effects of intravesical administration of paclitaxel (PTX-30W), which was prepared by solubilization with a water-soluble amphiphilic polymer composed of PMB30W, a copolymer of 2-methacryloyloxyethyl phosphorylcholine and *n*-butyl methacrylate, in an orthotopic bladder cancer model.

**Methods:**

The cytotoxicities of PMB30W were examined in MBT-2 cell cultures and the results were compared with those of the conventional paclitaxel solubilizer Cremophor. In an orthotopic MBT-2 bladder cancer model, the effect of intravesical administration of PTX-30W was compared with that of paclitaxel solubilized with Cremophor (PTX-CrEL). The paclitaxel concentration in bladder tumors after the intravesical treatment was also evaluated using liquid chromatography tandem mass spectrometry (LC-MS/MS) system.

**Results:**

*In vitro*, Cremophor exhibited dose-dependent cytotoxicity towards MBT-2 cells, whereas no cytotoxicity was observed with PMB30W. In the orthotopic bladder cancer model, intravesical administration of PTX-30W resulted in a significant reduction of bladder wet weight compared with that of PTX-CrEL. The paclitaxel concentration in bladder tumors after the intravesical treatment was significantly higher in PTX-30W treated mice than in PTX-CrEL treated mice.

**Conclusions:**

Intravesically administered PTX-30W can elicit stronger antitumor effects on bladder tumors than conventional paclitaxel formulated in Cremophor, presumably because of its better penetration into tumor cells. PTX-30W might be a promising antitumor agent for intravesical treatment of non-muscle invasive bladder cancer.

## Background

Among all newly diagnosed bladder cancer patients, 70% to 80% of patients are initially diagnosed as having non-muscle invasive cancers and undergo transurethral resection, which has a 70% local recurrence rate without any further adjuvant therapy [1,2]. To prevent recurrence, surgical resection is often followed by adjuvant intravesical immunotherapy or chemotherapy. As for bladder cancer, intravesical treatment is attractive. It is because direct delivery of therapeutic agent into bladder can achieve high concentration of drug at tumor sites while systemic drug uptake is limited.

Bacillus Calmette-Guérin (BCG) is currently the most successful agent for adjuvant intravesical treatment [3-6]. However, intravesical BCG therapy sometimes causes local or systemic side effects [7]. Furthermore, approximately 30% of patients still experience recurrence after BCG therapy [1]. Chemotherapeutic agents are also employed for intravesical treatment. Although the incidence of side effects is lower compared to BCG therapy, the recurrence rate is still high in intravesical chemotherapy [8]. A poor response to intravesical chemotherapy is thought to be in part due to the poor drug uptake into the bladder tissue within short indwelling times [9,10]. Intravesically administered drug is typically maintained in the bladder cavity for 2 hours. Moreover, in a clinical setting, in some patients, a drug can be held in the bladder for only a short time because of bladder irritability, and thus, intravesical treatment can be expected to be only slightly effective in such patients. Therefore, new agents which can be delivered more efficiently to the cancer tissue in the bladder are urgently needed.

Paclitaxel, originally derived from the bark of the Pacific Yew tree *Taxis brevifolia*, displays significant antineoplastic activity against various tumors, including bladder cancer [11,12]. Due to its poor aqueous solubility, Cremophor (polyoxyethylated castor oil) is used to dissolve paclitaxel for clinical use [13]. However, in some patients, Cremophor is often associated with side effects and adverse drug reactions [14,15]. Moreover, during intravesical treatment, Cremophor forms micelles in the bladder cavity and entraps paclitaxel into the micelles. This decrease in the free fraction of paclitaxel leads to lower drug penetration into the urothelial mucosa [16].

In this study, to overcome these problems, we used a polymer composed of 2-methacryloyloxyethyl phosphorylcholine (MPC) and *n*-butyl methacrylate (BMA) (PMB30W) as a solubilizer for paclitaxel. The PMB30W is comprised of a 30 mol% hydrophilic MPC unit and a 70 mol% hydrophobic BMA unit. Since the MPC unit is hydrophilic, PMB30W can be dissolved in water and form aggregate structure. Also thanks to the hydrophobic properties of the BMA unit, PMB30W can solubilize hydrophobic drugs such as paclitaxel. The solubility of paclitaxel in PMB30W aqueous solution is 1000-fold higher than that in water [17,18]. PMB30W is also characterized by its nontoxicity and its ability to penetrate quickly into exposed cells [19,20]. We hypothesized that the quick penetration of PMB30W into cells would lead to a higher drug concentration in bladder tumors and result in favorable cancer control in intravesical treatment.

In the present study, we employed an MBT-2 orthotopic mouse bladder cancer model which closely mimics the environments that exist in human bladder cancer. We previously demonstrated successful implantation of bladder tumors after instillation of MBT-2 cells into C3H/HeN mice with an incidence of almost 100% [21], and we have been using this novel MBT-2 orthotopic bladder cancer model to investigate the therapeutic effects of various intravesical agents [22-24].

In the present study, the cytotoxicity of blank vehicle (PMB30W) against MBT-2 cell line was evaluated *in vitro*. Then, in the orthotopic bladder cancer model, we evaluated the therapeutic effect of intravesically administered paclitaxel solubilized with PMB30W (PTX-30W) and examined the paclitaxel concentration in bladder tumors after intravesical administration of PTX-30W.

## Methods

### Chemicals

Paclitaxel powder was purchased from Wako Pure Chemical Industries (Osaka, Japan). Cremophor and docetaxel were purchased from Sigma-Aldrich (St. Louis, MO, USA). Paclitaxel solubilized with Cremophor (PTX-CrEL) was obtained from Bristol-Myers Squibb (Tokyo, Japan).

### Tumor cell line and animals

MBT-2, which was established from *N*-[4-(5-nitro-2-furyl)-2-thiazolyl]-formamide-induced urothelial carcinoma of the bladder of a female C3H/HeN mouse, was maintained in Roswell Park Memorial Institute (RPMI)-1640 medium containing 10% heat-inactivated fetal bovine serum at 37°C and 5% CO^2^.

Eight-week-old female C3H/HeN mice were purchased from Sankyo Laboratory Service Co (Tokyo, Japan). Mice were kept under standardized laboratory conditions with free access to food and water.

### Preparation of PTX-30W and PTX-CrEL

The PMB30W was synthesized by a previously described polymerization technique [18,25]. Fifty mg of PMB30W was dissolved in 1 ml of phosphate buffered saline (PBS). Five mg of paclitaxel dissolved in 100 μl of ethanol was then added to the PBS solution containing PMB30W. The ethanol was removed by evaporation under reduced pressure. The resulting paclitaxel concentration was 5.0 mg/ml. It was used following dilution with PBS. For the Cremophor formulation, paclitaxel was dissolved in Cremophor: ethanol (1:1, v/v) at a concentration of 6.0 mg/ml and used following dilution with PBS.

### LDH assay for determination of cytotoxicity of PMB30W and Cremophor

To examine the damage to the cell membrane caused by blank vehicles, lactate dehydrogenase (LDH) assay which is based on the leakage of LDH from cytosol was carried out. MBT-2 cells were seeded into 96-well plates at 1.0 × 10^4^ cells/well in 100 μl of RPMI-medium containing 5% fetal bovine serum. After preincubation for 24 hours, the medium was aspirated and fresh medium containing varying concentrations of PMB30W and Cremophor was added. Cells were exposed to the vehicles for 72 hours. Thereafter, 50 μL of cell-free supernatant was assayed using CytoTox 96 nonradioactive cytotoxicity assay (Promega, Madison, WI, USA) according to the manufacturer’s protocol, and damage to the cell membrane was determined. LDH activity was measured by determining the absorbance at 490 nm with a microplate reader. The percentage of LDH release was calculated using the following formula: percentage of release = 100 × (experimental LDH release – spontaneous LDH release)/(maximal LDH release – spontaneous LDH release). Spontaneous or maximal LDH release was determined in the presence of either medium alone or 1% Triton X-100, respectively. The experiments were repeated independently three times.

### Intravesical implantation of MBT-2 cells to establish an orthotopic bladder cancer model

All of the procedures involving animals and their care in this study were approved by the Animal Care Committee of Keio University in accordance with institutional and Japanese government guidelines for animal experiments. An orthotopic bladder cancer model was established by transurethral implantation of MBT-2 cells as previously described [21]. Briefly, the mice were anesthetized with an intraperitoneal injection of 1.5 mg/200 μl pentobarbital sodium. Urine was evacuated from the bladder by applying mild pressure on the abdomen. A 24-gauge Teflon-coated catheter was introduced into the lumen of the bladder through the urethra. MBT-2 cells, 5 × 10^5^ in a 50 μl suspension of serum-free RPMI-1640 medium, were then injected into the bladder. The catheter was removed, and to prevent voiding of the MBT-2 cells, the urethra was tied with 4-0 silk thread for 2 hours.

### Intravesical PTX-30W administration in an orthotopic bladder cancer model

The mice were randomly separated into three groups according to the agent for intravesical treatment (PBS, PTX-30W, or PTX-CrEL; n = 9 in each group). PBS was administered as a control. The total injection volume per animal was set to 50 μl and the exposure time was set to 30 minutes. The concentration of paclitaxel in PTX-30W and PTX-CrEL was fixed at 2 mg/ml. Intravesical drug administration was performed in the same manner as the tumor inoculation described above. Each treatment was repeated 6 times at 2-day intervals starting from day 4 after tumor implantation (on days 4, 7, 10, 13, 16, and 19). Previously our group has evaluated the pathological findings of bladder tumors in mice sacrificed 3 days after the instillation of MBT-2 cell suspension, and found the cells formed tiny superficial tumors, which gradually grew as apparent visible masses [21]. Therefore, we planned to start the intravesical treatment on day 4. On day 22, all mice were sacrificed and underwent necropsy. The bladder wet weights were measured to examine the growth of the bladder tumors.

### Measurement of paclitaxel concentration in bladder tumors

For the drug uptake study, another set of 22 mice were prepared and inoculated with MBT-2 cells in the bladder as described above. On day 20, the mice were divided into two groups, 12 mice administered PTX-30W and 10 mice administered PTX-CrEL. The concentration of paclitaxel in the mice administered PTX-30W and PTX-CrEL was 2 mg/ml and the total injection volume per animal was set to 50 μl. The mice were sacrificed 30 minutes after intravesical administration of the drugs. The bladder was removed from each mouse and the tumor tissue was harvested. The bladder tumors were vigorously washed three times in 20 ml PBS in order to remove any paclitaxel still attached to the tumor surface.

The paclitaxel concentration in the bladder tumors was analyzed using liquid chromatography tandem mass spectrometry (LC-MS/MS). The tumor tissues were weighed and homogenized in 1 ml methanol. The homogenate was centrifuged at 15000 g for 5 minutes and the supernatant was recovered as a sample. Volumes of 20 μl of the internal standard (docetaxel, 1000 ng/ml) and 2 ml of *tert*-butyl methyl ether were added to each sample. The samples were then thoroughly vortex mixed, followed by centrifugation at 1500 g for 5 minutes at 4°C. The supernatant was transferred and evaporated to dryness under a stream of nitrogen. The residue was reconstituted in 0.5 ml of methanol and used for analysis with the LC-MS/MS system. The LC-MS/MS system consisted of an UPLC system (Waters, Milford, MA, USA) and a mass spectrometer (API5000, AB/MDS Sciex, Framingham, MA, USA). A Cadenza CD-C18 (150 × 2.0-mm inner diameter; 3.0-μm particle size; Imtakt Corp, Kyoto, Japan) was used as the analytical column. Samples were eluted using a gradient at a flow rate of 0.2 ml/min. Mobile phase A was formic acid and mobile phase B was methanol. For the initial 3.5 minutes A was 25% and B was 75%, then B was increased to 90% for the next 3.5 minutes. After that, the initial condition of A 25% and B 75% was resumed for 2 more minutes for a total run time of 9 minutes. Paclitaxel was quantified by monitoring the ion transition of 854 → 286 *m/z*.

### Statistical analysis

Values are presented as the mean ± standard error, and data were analyzed by analysis of variance and the Mann-Whitney test. Statistical significance was determined at p < 0.05.

## Results

### Cytotoxicity of blank vehicles

**Figure 1:**
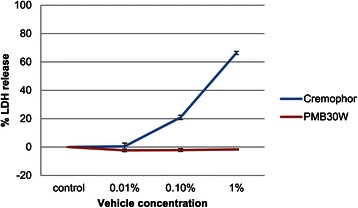
Cytotoxicity of blank vehicles. To determine the cytotoxicity of blank vehicles, MBT-2 cells were exposed to PMB30W or Cremophor for 72 hours. The level of LDH released into the culture supernatant from the cells was measured using LDH assay. PBS was used as control. The experiments were repeated independently three times. The data are shown as means ± S.E.

The cytotoxicity of the blank vehicles (PMB30W and Cremophor) was examined using an LDH assay. As shown in Figure 1, Cremophor exhibited dose-dependent cytotoxicity towards MBT-2 cells. In contrast, PMB30W showed no cytotoxicity. Even in concentrations up to 5%, PMB30W remained non-cytotoxic (data not shown) whereas Cremophor became too sticky to handle and place in the wells at a 5% concentration so the cytotoxicity could not be evaluated.

### Antitumor effect of intravesical PTX-30W and PTX-CrEL in the orthotopic bladder cancer model

**Figure 2:**
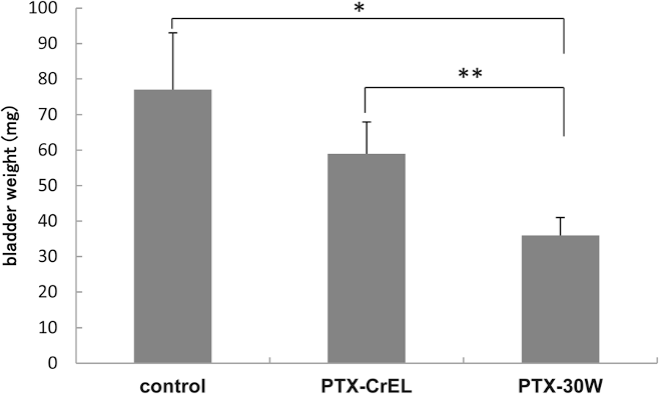
Bladder wet weights after intravesical treatment. Intravesical administration of PBS, PTX-30W or PTX-CrEL was repeated 6 times at 2 day intervals starting from day 4 after tumor implantation. On day 22, all mice were sacrificed and underwent necropsy. In the PTX-30W group, the mean bladder wet weight was 36 ± 5 mg, which was significantly lower than that in the control group (77 ± 16 mg; p = 0.0217) and PTX-CrEL group (59 ± 9 mg; p = 0.0469).

Twenty-seven mice were transurethrally inoculated with 5 × 10^5^ MBT-2 cells on day 0 and received intravesical instillation of control PBS, PTX-30W (2 mg/ml), or PTX-CrEL (2 mg/ml) on days 4, 7, 10, 13, 16 and 19. The mice were sacrificed on day 22 and the bladder wet weights were measured to examine the growth of the bladder tumors. As shown in Figure 2, intravesical administration of PTX-30W resulted in a significant reduction of bladder wet weight (36 ± 5 mg) as compared with those of the control group (77 ± 16 mg; p = 0.0217) and PTX-CrEL group (59 ± 9 mg; p = 0.0469). However, there was no significant difference in bladder wet weight between the control group and PTX-CrEL group.

### Paclitaxel uptake in bladder tumors

**Figure 3:**
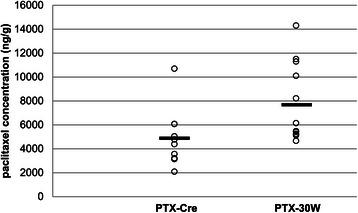
Paclitaxel concentration in bladder tumor after intravesical treatment. After intravesical administration of PTX-30W or PTX-CrEL, the paclitaxel concentration in bladder tumor was analyzed using LC-MS/MS. In the PTX-30W group, the paclitaxel concentration in the bladder tumors was 7719 ± 3274 ng/g, which was significantly higher than that in the PTX-CrEL group (4905 ± 2412 ng/g; p = 0.0147).

The paclitaxel uptake in bladder tumors after intravesical administration of paclitaxel formulation was evaluated. Twenty days after transurethral implantation of MBT-2 cells, PTX-30W (2 mg/ml) or PTX-CrEL (2 mg/ml) was administered transurethrally into the bladders of mice. After 30 minutes of administration, the mice were sacrificed and the amount of paclitaxel in the bladder tumor tissue was analyzed by LC-MS/MS. As shown in Figure 3, the paclitaxel concentration in the bladder tumors was significantly higher in the PTX-30W treated group than in the PTX-CrEL treated group (7719 ± 3274 ng/g vs 4905 ± 2412 ng/g; p = 0.0147), indicating that the penetration of intravesically administered PTX-30W into the bladder tumors was more efficient than PTX-CrEL.

## Discussion

To deliver paclitaxel to cancer tissue in the bladder, Cremophor is usually used to solubilize the paclitaxel, however, it can entrap the drug in micelles, thereby the drug penetration into the urothelial mucosa is limited [16]. To overcome this limitation of Cremophor, we employed PMB30W as the vehicle for the delivery of paclitaxel. The objectives of the present study were to evaluate the therapeutic effect of intravesically administered PTX-30W in an orthotopic bladder cancer model and to investigate the paclitaxel uptake into the bladder tumors.

In an *in vitro* study, we evaluated the cytotoxicity of the blank vehicles using LDH assay. Cremophor exhibited dose-dependent cytotoxicity against the MBT-2 cells whereas no cytotoxicity was observed with PMB30W. While the toxicity of Cremophor is not an issue with respect to cancer cells, it poses a problem in terms of patient safety since its severe side effects may injure normal urothelial mucosa, which of course is a concern. Cremophor contains castor oil which is considered to have cytotoxicity and a previous study demonstrated that intravesical administration of Cremophor caused inflammation in non-tumor bearing bladder tissue [26].

To overcome the limitations associated with using Cremophor for intravesical treatment, various devices were developed as solubilizers of paclitaxel in previous studies. Le Visage *et al.* [27] prepared polymethylidene malonate microspheres, which are characterized by the slow and continuous release of paclitaxel for intravesical treatment. However, the slow release of paclitaxel means that most of the encapsulated paclitaxel is voided before it can be taken up into bladder tissue. On the other hand, Lu *et al.* [28] reported the development of paclitaxel-loaded gelatin nanoparticles which were designed to release paclitaxel rapidly for intravesical use. However, the bladder tissue concentrations of paclitaxel after instillation of the nanoparticles were lower than that of free paclitaxel. Rapidly released paclitaxel seems to have been lost through the first urine void before they were taken up into the bladder tissue. These previous studies indicate that efficient drug uptake into bladder mucosa, even within a short drug indwelling time, is essential for intravesical chemotherapy.

To investigate the therapeutic effect of intravesically administered PTX-30W, we used an orthotopic bladder cancer model, which most closely mimics the clinical situation of intravesical tumors. Another advantage of such a model is that an immune-competent animal is used. To determine the exact antitumor effect induced by the treatment, immunologic reaction should be taken into consideration. Thus, a wild-type mouse cancer model must be used. Our MBT-2 orthotopic bladder cancer model using wild-type mice is considered to be a prerequisite for drawing a meaningful conclusion from our study.

In the present study, we employed the amphiphilic agent PMB30W as a carrier of paclitaxel. In a previous *in vitro* study in living cells, fluorescent-tagged PMB30W was observed to penetrate across the plasma membrane and entered the cytoplasm of the cells within a few minutes [20]. We investigated whether this characteristic of PMB30W would work advantageously in an orthotopic bladder cancer model. In the present orthotopic bladder cancer model, significant bladder tumor suppression was observed in the intravesical PTX-30W treated group compared with both the PBS control group and the PTX-CrEL group. Moreover, the uptake study showed significantly higher paclitaxel concentrations in bladder tumor tissues in the PTX-30W group than in the PTX-CrEL group 30 minutes after intravesical administration. These results seem to indicate that the superior antitumor effect of PTX-30W in orthotopic bladder tumors is attributed to its efficient uptake into tumor tissue following intravesical administration. Therefore, PTX-30W was considered to be suitable for intravesical administration.

Soma *et al.* [29] evaluated the efficacy of intraperitoneally administered PTX-30W in a peritoneal metastasis model of gastric cancer in nude mice. They demonstrated that intraperitoneal administration of PTX-30W could suppress peritoneal metastasis more effectively than PTX-CrEL, presumably because of its better penetration into the disseminated peritoneal tumors. Their study findings are in agreement with those of the present study with regards to the point that PTX-30W is superior in intracavitary treatment in which the administered agents can be delivered directly at a high concentration to the tumor lesion, however, what makes our study different from theirs is the drug exposure time. While intraperitoneally administered drug is allowed to contact tumor tissues until it is absorbed from the cavity, intravesically administered drug can contact tumor tissues only until it is flushed out from the cavity by urination. Because of the disadvantage of the short exposure time, multiple instillations of PTX-30W were planned in the first set of experiment and the results showed that the intravesically administered PTX-30W could significantly suppress bladder tumor growth and exhibited better drug uptake compared with PTX-CrEL. One limitation of the present study is that we did not evaluate the correlation of the tumor concentration of PTX-30W with tumor size. However, we believe that a high drug concentration after a single instillation of PTX-30W had an effect on the antitumor activity of the drug.

## Conclusion

We have shown that intravesical PTX-30W treatment exhibits significant tumor suppression in an orthotopic bladder cancer model compared to conventional PTX-CrEL and that efficient penetration of the PTX-30W into tumor tissue seemed to be associated with its antitumor effect. The results of the present study indicate that intravesical PTX-30W treatment may be a promising new therapy for non-muscle invasive bladder cancer.

## Abbreviations

LC-MS/MS: Liquid chromatography tandem mass spectrometry
PBS: Phosphate buffered saline
LDH: Lactate dehydrogenase
